# Barriers to Maternal and Child Health Care Service Uptake in Assosa Zone, Benishangul Gumuz Region, Ethiopia: A Qualitative Study

**DOI:** 10.1155/2021/5154303

**Published:** 2021-11-06

**Authors:** Mulatu Agajie, Solen Abera, Eshetu Yimer, Gizachew Yaregal, Amir Muhidin, Wagari Kelbessa, Dula Ayana, Debebe Shaweno

**Affiliations:** ^1^Assosa University, College of Health Science, Department of Public Health, Ethiopia; ^2^Benishangul Gumuz Regional Health Bureau, Ethiopia; ^3^Assosa General Hospital, Ethiopia; ^4^School of Health and Related Research, University of Sheffield, Sheffield, England, UK

## Abstract

**Background:**

Ethiopia has reduced maternal mortality from 871 to 412 per 100,000 live births between 2000 and 2016. In 2019, under-5 mortality rates in Ethiopia were 55 deaths per 1,000 live births. Benishangul Gumuz was the second-largest region in the under-5 mortality rate (98/1,000 live births) in the country. Maternal and child health care service uptake is an important indicator of health outcomes. This study is aimed at exploring major barriers to maternal and child health care uptake in Assosa Zone.

**Methods:**

This study was conducted in the Bambasi, Menge, and Sherkole districts of the Assosa Zone from July 17 to August 31/2019. The study explored the life experience of study participants about MCH services. The sampling technique was purposive, and data collection methods were focus group discussions, key informant interviews, and in-depth interviews. Data were analyzed thematically.

**Result:**

The main barriers to child health care services were financial problems, lack of knowledge, preference of traditional medicines for a sick child, women having no time to care for their sick child, poor roads. poor health facility readiness, the poor economy of families, lack of ambulance, cultural and traditional beliefs, providers being male, and unprofessional behaviors which were the major barriers hindering the uptake of maternal health service utilization.

**Conclusion:**

Poor health facility readiness, indirect costs, inaccessibility to health facilities, and cultural and traditional practices were among the major barriers to service uptake identified by this research in the study area.

## 1. Introduction

The global maternal mortality ratio has decreased by 44% between 1990 and 2015 [1]. But, still, now, hundreds of thousands of mothers are dying from easily preventable causes [2]. Globally, the underfive mortality rate dropped to 41 deaths per 1,000 live births in 2016 from 93 in 1990, a 56 percent decline [3]. Maternal and child healthcare service uptake is an important indicator of the health outcomes of mothers and children [4]. Ethiopia has also reduced maternal death from 871 to 412 between 2000 and 2016 [5]. Between 2005 and 2019, Ethiopia has reduced the underfive mortality rate from 123 to 55 deaths per 1,000 live births, the infant mortality rate from 77 to 43 deaths per 1,000 live births, and neonatal mortality rate from 39 to 30 deaths per 1,000 live births [6]. Ethiopia has to do much more to reach the goal of reducing the global maternal mortality ratio to less than 70 per 100,000 live births, neonatal mortality rate to less than 12 per 1,000 live births, and underfive mortality rate to less than 25 per 1,000 live births [7]. The reduction of maternal, neonatal, infant, and underfive mortality rates is by large attributable to the utilization of promotive, preventive, and curative primary health care interventions [8]. According to Ethiopian mini DHS 2019, coverage of ANC 4+ visit was 43%, skilled delivery was 50%, PNC in the first two days after delivery was 34%, and modern contraceptive use was 41%, which indicates even though maternal health services are available free of charge, women are not motivated to use the services. There is much variability between regions of Ethiopia; Benishangul Gumuz Region is the second highest in underfive mortality which is 98 deaths per 1,000 live births [5]. Barriers to health care services have many impacts on maternal and child health and survival, particularly in rural settings where there is inadequate access or poor utilization of health services. Therefore, exploring major barriers to maternal and child health care uptake in the study area could inform program managers about where to focus interventions that can reduce maternal and newborn mortality and improve their health outcomes.

## 2. Materials and Methods

### 2.1. Study Area and Period

A qualitative study was conducted in rural kebeles of the Bambasi, Menge, and Sherkole districts of the Assosa Zone in the Benishangul Gumuz Region between July 17 and August 31/2019. The study population was women development army members, (clan, religious, and kebele) leaders, and health extension workers, health workers working in underfive clinics, and mothers of children with chronic and acute illnesses. The FGDs were held separately for both sexes.

### 2.2. Study Population

The Sampling technique was purposive, and data collection methods were focus group discussions (FGD), key informant interviews (KII), and in-depth interviews (IDI). A total of 15 FGDs, 8 KII, and 9 IDI were conducted. One hundred sixty-seven participants have participated in the study. Data were analyzed using a thematic analysis approach. Focus group discussions were held among members of the women's development army, clan, kebele, and religious leaders. Key informant interviews were held with health extension workers and among health care workers in health centers and in-depth interviews with women whose children had chronic illnesses. Idea saturation for our study was reached after interviewing 150 FGD participants, 8 key informant interviewees, and 9 in-depth interview participants.

### 2.3. Research Instruments and Data Collection

FGD was held separately for both sexes using the guideline. In-depth interviews and KII were also conducted using interview guides to collect the data. During focus group discussions, one person facilitated the discussion whereas the other took important notes and tape-recorded them.

### 2.4. Data Quality and Analysis

Data collectors were selected based on their experience in qualitative research and training in the data collection process. At the end of every day, a debriefing session between data collectors was done. The interviews were conducted in the participant's local language and translated to English; the researcher was fluent in both languages. All interview records were transcribed verbatim. The guides for interviews and FGD were pretested on 5 percent of the total samples outside the actual data collection sites before actual data collection, and amendment was done accordingly. The objective of the research was clearly described to respondents before taking the interviews. The audio voice was recorded from the respondents and finally transcribed. Mixed methods (FGD, IDI, KII, and observation) among different participants for data collection were used to cross-verify the collected data. Supervision meetings and discussions were also conducted to authenticate collected data. Health facility readiness assessment tools with the parameters, facility staffing, infrastructure, availability of transportation for emergencies, communication, power supply, source of water, infection prevention, processing equipment for use, waste disposal system, supervision, general services, and structure and availability of basic supplies were assessed using standard checklists to determine whether the facilities had fulfilled the minimum standard requirements. One hundred thirty study participants have claimed that superstitions are responsible for not availing the available health care facility. Themes were identified after reading and rereading the data, and finally, the analysis was performed thematically.

### 2.5. Ethical Considerations

Ethical clearance was obtained from the ethical review committee of Assosa University. Participants were informed about the objective of the study and the right to refuse or withdraw from the study at any time. Audio recordings and transcripts were kept confidential and codes were used instead of names. Before the interview, informed and verbal consent was obtained from the participants.

## 3. Results

A total of fifteen focus group discussants, eight key informant interviewees, and nine in-depth interviewees have participated in the study. The age of study participants has ranged from 24 to 63 years.

When asked what actions they take when children get sick; the majority of FGD participants said that despite many barriers they are facing at the health facilities, they still go to health facilities for treatment. Almost all participants across all districts indicated timely care-seeking for a sick child in their community which was reported to be a big challenge because of financial problems, remoteness of health facility, preference of traditional medicine over modern medicine, and women's workload, the traditional belief that the illness is due to evil eyes, and unavailability of basic medical supplies. The participants also agreed low utilization of child health care services because of the knowledge gap in the community about the importance of preventive child health care.

When asked whether childhood and newborn illness treatment was available at the community level, almost all participants from FGD and IDI revealed that there is no treatment for sick children and newborns in their communities.

*“The health care providers are not responsible and not respecting us, even when we request an ambulance, they always reply no fuel.”* (Woman, FGD, 35 years).

Another FGD participant said that *“Few mothers do not want to take their sick child to a health facility because diseases like malnutrition are due to evil eyes and health care providers do nothing for them and they believe taking them to traditional healer would be better. This is why malnutrition in children in our community became a huge problem. They do not accept the advice we give them.”* (Woman, FGD, 40 years).

*“I was dissatisfied with the care given to my sick child in the nearest health center because; there is no drug, no diagnostic materials, and no committed health workers and long waiting time.”* (Woman, IDI, 26 years).

*“….within the last five years, death due to malnutrition has occurred, mostly the community considers malnutrition as a punishment of sin from the Lord and believed to be due to evil eyes and they do not take malnourished children to a health facility for treatment. The other reason that makes malnutrition common is that most mothers are passing their time on gold mining leaving their children at home meanwhile no one cares and no food for these children.”* (Menge, KII, HEW).

All FGD participants across the three districts agreed on the importance of antenatal care for mother and child health; however, the timing to make the first visit was usually taken as a norm to be after five months of pregnancy and missing the appointments.

As reported by one of the KII:

*“Almost all farming and gold mining in our community are undertaken by women; farmland and mining sites are far away from home, they go in the morning and get back home in the evening and they miss their appointment days. The other challenge is that villages are far from the health center and the roads are not good, especially during the rainy seasons it is not functional. In addition, most of them do not have money to pay for transportation.”* (Menge, HEW, KII, 29 years).

Almost all FGD participants and KII reflected the importance of skilled delivery and prefer to deliver at a health facility. In contrast to this, most births occur at home because of different barriers: remoteness of the villages from the health facility, disrespect and uncaring providers, and unavailability of foods and drinks at health facilities for delivered mothers.

One of the FGD participants said

*“The reason for the very poor quality of services is that delivery attendants have no skills and knowledge and when they are asked for help they usually humiliate us. Some are not disciplined, they are making women's critical situations as a point of discussion and enjoyment which is unacceptable and discouraging. When women hear this information, they do not want to go to a health facility for delivery; they prefer to deliver at home where they are being respected.”* (Sherkole woreda, clan leader, FGD, 45 years).

Another FGD participant stated *“….Delivering at a health facility is very good but the most barriers for mothers are that many women who come from remote and stay longer do not get food and drinks at health facilities especially “Muk” which is important for mothers immediately after delivery; why not the government provide foods and drinks for laboring mother at a health facility?* (Bambasi, woman, FGD, 42 years).

Another key informant said

*“Mostly women complain that the health centers have no waiting rooms, where women stay if they come to health center during labor time. The reasons for the absence of waiting room for delivery are lack of budget and attention to this problems. Another reason why women deliver at home is that women fear to be attended by male midwives.”* (Sherkole health center, KII).

Another key informant said

*[1] “….lack of transport, lack of support from husband, economic problems, lack of ambulance service; midwives being male were the major reasons for very low institutional delivery. Women are encouraged by the elders to deliver at home. Elders convince them as if they safely deliver at home than at health facility.”* (Sherkole HEW).

Regarding postnatal care service uptakes, participants of FGD and KII across the three districts agreed that postnatal care is the less utilized MCH service in all communities and considered it not important. Women are not allowed to go outside the home 45 days after delivery, because culturally, it is believed that women face great health problem and she may bleed if she moves outside and may die.

*“…our communities culturally do not allow women to move outside the home 45* days *after delivery. And we health care providers also do not provide PNC services at home because usually, the health center did not assign providers for it.”* (Menge, KII, HW).

All FGDs and KIIs participants reported that family planning is also a less utilized maternal health service in their respective communities. Culturally, women are not allowed to use family planning because of strong male dominance and opposition and taken to be as opposing to what Allah says to humans. Usually, children are seen as an asset in the community. One key informant said that

*“Family planning is less utilized services, because of husband opposition and it is forbidden to take family planning, visible implant. Women who took implants are not allowed to enter in mosques.”* (Sherkole, HEW).

In all communities, poverty was reported to be the primary barrier to timely seek treatment for children. Participants indicated that the decision-making power of MCH services is dominantly made by husbands. Money is controlled exclusively by husbands.

One member of the participants said

*“When at home; we abuse our wife's right using family planning but at the meeting, we do not tell the fact, we mostly describe as if we allow our wife to use family planning.”*

(Menge, clan leader, FGD, 45 years).

Another member of the participants said that

*“…….our husbands are supporting us in many ways, except opposing family planning use. Women do not take any family planning without the willingness of their husbands, even it is difficult to request about it.”* (Menge, woman, FGD, 46 years).

All participants reported remoteness to the health facilities, unavailability of an ambulance, lack of basic supplies, poor compassion, caring, and respectful providers, and long waiting time at health facilities which were the major barriers that affect service utilization of mothers and their children.

## 4. Discussion

This study was aimed at exploring barriers to maternal and child health care service utilization in three districts of Assosa Zone, Benishangul Gumuz Region, Ethiopia. Health care service uptake during pregnancy, delivery, and postpartum period has significant importance for the survival and health of both mother and child. Despite these services are being provided free, the majority of women are not using them. Study participants have identified various barriers to maternal and child health care service uptake.

In the current study, child's health care service uptake from public health facilities was hindered by poor health-facility basic supplies, dependence on traditional medicine, traditional beliefs, inadequate public transport, and poor roads, low economic status of families, women's workload, and poor ICCM and CBNC implementation at the community level. The traditional belief at the community level was identified as an important barrier to timely accessing health care service uptake in the current study. Child health care services are poorly utilized if it has financial expenses such as buying drugs from private clinics and transportation costs and might delay or prevent poor families to seek care and can increase the risk of dying and severity of the disease. Similar findings were reported in Burkina Faso [9] and Ghana [10]. Another barrier was elders were perceived to be unsupportive to seek treatment for sick children from health facilities. The ICCM/CBNC services at the community level were also not fully functional, and the participants knew little about the services. These findings are consistent with previous studies in low- and middle-income countries [11], Malawi [12], and Wolkayit [13].

In this study, the knowledge about the importance of ANC was reported to be good, but the importance of making the first ANC early and visiting all ANC appointments is inadequate. Distant health facilities, women's heavy workload, the long waiting time to receive care, and limited transport availability appeared as a reason for late and missed ANC utilization. In line with these findings, studies conducted in Nepal [14] and South Sudan [10, 15] showed women often did not have enough time to visit a health facility due to their workloads. Another study conducted in Sierra Leone [16] revealed ANC was not timely due to lack of transport, distant health facilities, and social norms to delay care seeking until pregnancy is visible. Other similar findings were reported from Ghana [17], rural Tanzania [18], Malawi [19], and Senegal [20].

The Ethiopian government has subsidized MCH services to make them affordable, acceptable, and available. Despite these initiatives, our findings showed that the quality of services, providers being male, unavailability of foods and drinks, lack of ambulance, inconvenient waiting rooms, long-distance from health facilities, and unprofessional behaviors were among many barriers that limited women's access to skilled delivery services. The health care providers should respect the reproductive rights of laboring mothers such as keeping their confidentiality, providing care with respect and dignity, and the service should be mother-friendly services. Similar findings were reported from Sidama Zone [21], selected towns in Ethiopia [22], and Ethiopia [23]. Physical abuse including slapping at the delivery bed was also reported in Nigeria [24]. A study from Eastern Nepal [25] reported as laboring mothers were not comfortable exposing their body parts to male providers. Most pregnant women have taken home as a natural place of delivery where they could get adequate foods and drinks, where they are being emotionally supported by family members and being respected. This finding is consistent with a study in rural areas of Nepal [14], preference of home delivery for the wish to be cared for by family members, greater freedom of movement at home, and the possibility to obtain appropriate “hot” foods.

Currently, in Ethiopia, only 50% of women get assistance from skilled providers, whereas half have no access to skilled care, which increases the risk of morbidity and mortality to mothers [26].

The Ethiopian government has taken encouraging measures in availing ambulances for laboring mothers to move them timely to health facilities; however, participants in this particular study reported very limited availability of ambulances for laboring women. This finding revealed the demand for skilled delivery at a health facility, but unavailability of an ambulance and/or fuel and lack of alternative transportation means during labor and emergency could delay or prevent from going to the health facility. This finding is supported by a study done in rural Ghana [17] that demonstrated that distant health facilities and lack of ambulances play a significant role in reduced skilled delivery service utilization.

PNC was reported to be the less utilized maternal health service. Even though there was a difference among districts, PNC was reported to have no advantages unless mothers face health problems. This study also revealed that culturally, women were not allowed to move outside the home 45 days after delivery which was perceived to expose mothers to bleeding. Thus, most participants had poorly understood about complications of mothers and newborns after delivery. To reduce neonatal mortality, health extension workers were expected to provide postnatal care services after delivery through home visiting. Despite the importance of PNC visits, all women were not receiving PNC at home or health facilities because of HEW availability and performance. This finding is also supported by the study conducted in the Wolkaiye Tigray region [13], rural areas of Nepal [25], selected towns of Ethiopia [22], and Sierra Leone [16].

The main barriers to family planning service uptake reported by the participants were a strong cultural desire for large family size (perception of children as an asset), male opposition and domination, and religious restriction. Our findings are consistent with studies conducted in Burundi and Northern Uganda [27], Bale [28], and Rwanda [26], which have identified a lack of decision-making powers of women or husband domination and opposition and desire for a larger family as the reasons for not using family planning. This study showed an association between religious beliefs and family planning; it is forbidden to have implants and enter into the mosque. Consistent with our study in rural Tanzania [29] and South Africa [30], it showed religious and cultural norms as a barrier to family planning utilization.

## 5. Conclusion and Recommendations

The barriers identified gave us a clue why reducing maternal and newborn death in the region is a serious challenge for the government. Poor health facility readiness, indirect costs, inaccessibility to health facilities, and cultural and traditional practices were among the major barriers to service uptake identified by this research in the study area. Health facility readiness must be given due attention to improving the quality of maternal and newborn care. The practical solution must be considered for practical barriers to maternal and child health service utilization in the region. The government should address geographic access problems and transportation barriers. Different partners and stakeholders should do strong awareness creation and community mobilization programs about women empowerment through involving religious and clan leaders, traditional healers, and other relevant bodies to increase the uptake of MCH services in the region.

## Data Availability

The data used to support the finding of this study are available from the corresponding author upon formal request.

## Abbreviations

CBNC: Community-Based Newborn Care
FGD: Focus group discussion
HEW: Health extension workers
HW: Health worker
ICCM: Integrated community case management
IDI: In-depth interview
KII: Key informant interview
KOICA: Korea International Cooperation Agency
MCH: Maternal and child health.
